# Physical activity among children and adolescents in Germany. Results of the cross-sectional KiGGS Wave 2 study and trends

**DOI:** 10.17886/RKI-GBE-2018-023.2

**Published:** 2018-03-15

**Authors:** Jonas D. Finger, Gianni Varnaccia, Anja Borrmann, Cornelia Lange, Gert B. M. Mensink

**Affiliations:** Robert Koch Institute, Berlin, Department of Epidemiology and Health Monitoring

**Keywords:** PHYSICAL ACTIVITY, MOVEMENT GUIDELINES, CHILDREN AND ADOLESCENTS, HEALTH MONITORING, KIGGS

## Abstract

Self-reported data from wave 2 of the German Health Interview and Examination Survey for Children and Adolescents (KiGGS Wave 2, 2014-2017) provides the basis for assessing whether the levels of physical activity of children and adolescents in Germany meet the levels recommended by the World Health Organization (WHO). Merely 22.4% of girls and 29.4% of boys in the 3-17 age group are physically active for at least 60 minutes per day and therefore meet the WHO recommendations. Prevalence of recommended levels of physical activity decreases continuously with age, both for girls and boys. In KiGGS Wave 2, girls in the 3-10 age group met the levels of physical activity recommended by the World Health Organization significantly less often than in KiGGS Wave 1. Low levels of physical activity were highest amongst adolescent age girls, as well as among boys and girls of low socioeconomic status. The results indicate a great potential to promote physical activity.

## Background

Physical activity is defined as any kind of movement by skeletal muscles that leads to an increased use of energy [1]. According to the Global Burden of Disease Study 2016 [2], lack of physical activity in Germany is behind 12.3% of coronary heart disease, 7.6% of stroke, 3.1% of diabetes mellitus, 3.4% of colorectal cancer and 1.8% of breast cancer deaths. Participation in school sports and higher levels of physical activity during leisure time is moreover linked to a lower risk of mental illnesses [3]. Promoting physical activity at child and adolescent age can contribute towards prevention of obesity [4, 5] and attention deficit/hyperactivity disorder [6], promotion of healthy development [7] and greater cognitive and academic achievements [8], as well as increased levels of physical activity at adult age [9]. The WHO’s ‘Global action plan on physical activity 2018-2030’ also highlights the particular importance of childhood and adolescence for the promotion of physical activity [10]. The Action Plan contains specific recommendations for action to achieve the ‘voluntary global target’ of reducing the prevalence of insufficient levels of physical activity by 10% between 2010 and 2025 [11]. According to the WHO definition, children and adolescents should achieve at least 60 minutes of moderate- to vigorous-intensity physical activity daily [12]. According to the German Health Interview and Examination Survey for Children and Adolescents 2009-2012 (KiGGS Wave 1), 25.4% of girls and 29.4% of boys aged 3-17 meet the levels recommended by the WHO [13]. This article presents the surveyed prevalence of physical activity among children and adolescents in Germany in KiGGS Wave 2 and compares it to the levels reported in KiGGS Wave 1.

**KiGGS Wave 2**

Second follow-up to the German Health Interview and Examination Survey for Children and Adolescents

**Data owner:** Robert Koch Institute

**Aim:** Providing reliable information on health status, health-related behaviour, living conditions, protective and risk factors, and health care among children, adolescents and young adults living in Germany, with the possibility of trend and longitudinal analyses

**Study design**: Combined cross-sectional and cohort study

**Cross-sectional study in KiGGS Wave 2**

**Age range:** 0-17 years

**Population:** Children and adolescents with permanent residence in Germany

**Sampling:** Samples from official residency registries - randomly selected children and adolescents from the 167 cities and municipalities covered by the KiGGS baseline study

**Sample size:** 15,023 participants

**KiGGS cohort study in KiGGS Wave 2**

**Age range:** 10-31 years

**Sampling:** Re-invitation of everyone who took part in the KiGGS baseline study and who was willing to participate in a follow-up

**Sample size:** 10,853 participants

**KiGGS survey waves**

► KiGGS baseline study (2003-2006), examination and interview survey
► KiGGS Wave 1 (2009-2012), interview survey
► KiGGS Wave 2 (2014-2017), examination and interview survey

More information is available at www.kiggs-studie.de/english

## Indicator and Methodology

KiGGS forms part of the health monitoring program undertaken at the Robert Koch Institute and includes repeated cross-sectional surveys of children and adolescents aged between 0 and 17 years that are representative of the German population (KiGGS cross-sectional study). After having carried out the baseline study as an interview and examination survey between 2003 and 2006, and KiGGS Wave 1 as an interview-based survey between 2009 and 2012, KiGGS Wave 2 was implemented between 2014 and 2017 as a combined interview and examination survey.

A detailed description of the methodology used in KiGGS Wave 2 can be found in New data for action. Data collection for KiGGS Wave 2 has been completed in issue S3/2017 as well as KiGGS Wave 2 cross-sectional study – participant acquisi tion, response rates and representativeness in issue 1/2018 of the Journal of Health Monitoring [14, 15].

In KiGGS Wave 2 physical activity data was self-reported (11-17 age group) or provided by parents and legal guardians (3-10 age group) via a self-administered questionnaire. Participants were asked, ‘On how many days of a normal week are you/is your child physically active for at least 60 minutes on a single day?’ The eight answer categories ranged from ‘On no day’ to ‘On seven days’. Based on this data, the survey assessed whether interviewees met the WHO recommended ‘at least 60 minutes of moderate- to vigorous-intensity physical activity daily’ [12]. An indicator for ‘low levels of physical activity’ was created for those who engage in physical activity of at least 60 minutes per day on less than two days per week. A trend for the recommended levels of physical activity can only be established between KiGGS Wave 1 and 2, as the question to survey physical activity was the same in both waves and an analogous indicator can therefore be calculated.

The analysis is based on the data received from 12,981 children and adolescents (6,532 girls and 6,449 boys) aged 3 to 17 with valid responses on physical activity. The results are presented as prevalences (expressed as percentages) and stratified by gender, age and socioeconomic status (SES) [16].

The calculations were conducted applying a weighting factor that corrects deviations within the sample from the German population with regard to age, gender, federal state, nationality and the parents’ level of education (Microcensus 2013 [17]).

The calculation of trends between KiGGS Wave 1 and 2 is based on weighted age-standardised prevalence (age and gender by population structure as of 31 December 2015). Logistic regression (t-Test) was used to test the statistical relevance of developments over time. This article reports the prevalence with 95% confidence intervals (95% CI). A statistically significant difference between groups is assumed to have been demonstrated with p-values of less than 0.05 (once weighting had been applied and the survey design had been taken into account).

## Results

**Figure 1:**
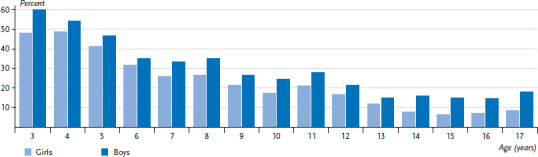
Prevalence of ‘at least 60 minutes of physical activity daily’ (‘WHO recommendation achieved’) according to age (n=6,532 Girls, n=6,449 Boys) Source: KiGGS Wave 2 (2014-2017) WHO: World Health Organization

KiGGS Wave 2 results indicate that fewer girls (22.4%) meet the recommendations on physical activity than boys (29.4%, Table 1). Gender differences are particularly marked in the 14-17 age group (Figure 1). With increasing age girls and boys achieve the recommended levels of physical activity less often. For boys, no link between recommended levels of physical activity and SES is apparent. The result for girls is inconsistent.

Girls (11.1%) more frequently show low levels of physical activity compared to boys (7.0%, Table 2). Prevalence of low levels of physical activity increases significantly in the 14-17 age group and is twice as high for girls compared to boys. Prevalence of low levels of physical activity is significantly higher for girls and boys with low SES compared to those with medium and high SES.

**Figure 2:**
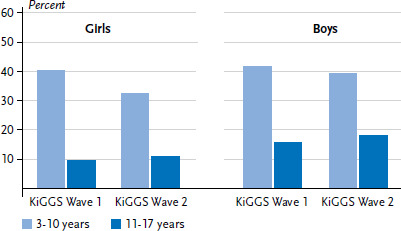
Trend for prevalence of ‘at least 60 minutes of physical activity daily’ (‘WHO recommendation achieved’) between KiGGS Wave 1 and KiGGS Wave 2 according to age (n=11,526 girls, n=11,518 boys) Source: KiGGS Wave 1 (2009-2012), KiGGS Wave 2 (2014-2017) WHO: World Health Organization

For girls, weighted and age-standardised prevalence of recommended levels of physical activity decreased significantly (from 25.9% to 22.4%) between KiGGS Wave 1 (2009-2012) and KiGGS Wave 2 (2014-2017). For boys the same prevalence did not change during this time (29.7% and 29.4%, data not shown). Figure 2 shows the trend between KiGGS Wave 1 and 2 by age groups. Decreasing prevalence among girls is related to the significant decrease in prevalence in the 3-10 age group (from 40.7% to 32.6%, Figure 2). Age-standardised prevalence of low levels of physical activity have increased significantly between KiGGS Wave 1 and 2 – the prevalence rose from 8.0% to 11.1% for girls and 4.6% to 7.0% for boys (data not shown).

## Discussion

KiGGS Wave 2 reveals similar patterns for the relationship between achieving the recommended levels of physical activity and gender, age and SES as observed in KiGGS Wave 1 [13].

The secular trend, in terms of the slight decrease of prevalence for girls to meet the recommended levels of physical activity between KiGGS Wave 1 and 2 is in line with the results of the WHO’s survey Health Behaviour in School-aged Children (HBSC). Between 2010 and 2014, the prevalence of girls who meet the recommended levels of physical activity decreased slightly in Germany, while the prevalence among boys remained unchanged [18]. When interpreting this trend, it is important to consider that the survey method changed between KiGGS Wave 1 (telephone interview) and KiGGS Wave 2 (self-administered questionnaire). We cannot exclude the possibility that social desirability led to an overestimation of the reported levels of physical activity in KiGGS Wave 1.

To calculate the indicator regarding the achievement of the WHO recommendations on physical activity, the survey used self-reported total physical activity, which included sports, as well as routine daily activities. Sports activities often included aerobic endurance activities, a type of activity explicitly commended by the WHO recommendations on physical activity due to their particularly beneficial effects on health [12]. Further KiGGS cohort-based [19] analyses indicate that children and adolescents from high SES family backgrounds are more frequently physically active members of sports clubs [13] and show higher levels of aerobic fitness [20] than children and adolescents from low SES family backgrounds. Keeping up physical activity from childhood to adolescence thereby depends on a number of family, health, behaviour and social environment factors [21], which need to be considered when planning measures to promote physical activity at child and adolescent age. Germany’s national health target ‘Grow up healthy’ includes promoting physical activity and is also supported by IN FORM – Germany’s national initiative to promote healthy diets and physical activity. Over three quarters of girls and two thirds of boys do not achieve the WHO’s recommended levels of physical activity, pointing to the great need for measures that promote physical activity. WHO physical activity guidelines are only a minimum recommendation, any physical activity beyond these levels can provide additional health benefits. Recognition of this fact is reflected in Germany’s National Recommendations for Physical Activity and Physical Activity Promotion that recommends at least 180 minutes of daily physical activity for children at Kindergarten and 90 minutes of daily physical activity for primary school aged children and adolescents, as well as a general reduction of the amount of time spent sitting [22]. Promoting the physical activity of children and adolescents should follow a settings-based approach and include measures to make Kindergartens, schools and the places where children and adolescents live, more movement-friendly. This should include health-oriented city planning, the reduction of risks and environmental pollution from road traffic, expanding foot and bike paths as well as child- and adolescent-friendly design of parks and recreation and sports facilities [22].

## Key statements

- Merely 22.4% of girls and 29.4% of boys in the 3-17 age group meet the physical activity recommendations of the World Health Organization.
- The share of children and adolescents who meet the physical activity recommendations of the World Health Organization decreases continuously with age.
- Prevalence of low levels of physical activity increases significantly with age and is twice as high for girls in the 14-17 age group than for boys.
- In KiGGS Wave 2, girls in the 3-10 age group meet the levels of physical activity recommended by the World Health Organization significantly less often than in KiGGS Wave 1.

## Figures and Tables

**Table 1:** Prevalence of ‘at least 60 minutes of physical activity daily’ (‘WHO recommendation achieved’) according to gender, age and socioeconomic status (n=6,532 girls, n=6,449 boys) Source: KiGGS Wave 2 (2014-2017) WHO: World Health Organization

Girls	Prevalence (%)	(95% CI)	Boys	Prevalence (%)	(95% CI)
**Girls (total)**	**22.4**	**(20.9-24.0)**	**Boys (total)**	**29.4**	**(27.6-31.2)**
**Age**			**Age**		
3-6 Years	42.5	(39.0-46.0)	3-6 Years	48.9	(45.2-52.6)
7-10 Years	22.8	(20.1-25.8)	7-10 Years	30.0	(27.1-33.1)
11-13 Years	16.5	(14.1-19.1)	11-13 Years	21.4	(18.7-24.3)
14-17 Years	7.5	(6.0-9.2)	14-17 Years	16.0	(13.8-18.6)
**Socioeconomic status**			**Socioeconomic status**		
Low	25.2	(21.5-29.4)	Low	31.1	(26.7-35.9)
Medium	20.8	(19.3-22.4)	Medium	28.6	(26.6-30.7)
High	24.4	(21.5-27.5)	High	30.6	(27.9-33.4)
**Total (Girls and Boys)**	**26.0**	**(24.7-27.4)**	**Total (Girls and Boys)**	**26.0**	**(24.7-27.4)**

CI=confidence interval

**Table 2:** Prevalence of ‘60 minutes of physical activity on less than two days per week’ (‘low levels of physical activity’) according to gender, age and socioeconomic status (n=6,532 girls, n=6,449 boys) Source: KiGGS Wave 2 (2014-2017)

Girls	Prevalence (%)	(95% CI)	Boys	Prevalence (%)	(95% CI)
**Girls (total)**	**11.1**	**(9.9-12.4)**	**Boys (total)**	**7.0**	**(6.2-8.0)**
**Age**			**Age**		
3-6 Years	6.7	(5.1-8.6)	3-6 Years	5.8	(4.4-7.6)
7-10 Years	5.7	(4.4-7.4)	7-10 Years	4.4	(3.2-6.1)
11-13 Years	8.4	(6.6-10.8)	11-13 Years	6.7	(5.0-9.0)
14-17 Years	22.0	(19.2-25.0)	14-17 Years	10.8	(8.7-13.5)
**Socioeconomic status**			**Socioeconomic status**		
Low	19.4	(15.8-23.6)	Low	11.6	(8.6-15.5)
Medium	9.6	(8.3-11.1)	Medium	6.3	(5.3-7.4)
High	7.6	(6.2-9.4)	High	4.4	(3.3-5.8)
**Total (Girls and Boys)**	**9.0**	**(8.3-9.8)**	**Total (Girls and Boys)**	**9.0**	**(8.3-9.8)**

CI=confidence interval
