# Corrigendum: Physical activity among children and adolescents in Germany. Results of the cross-sectional KiGGS Wave 2 study and trends

**DOI:** 10.25646/6800

**Published:** 2018-04-12

**Authors:** JD Finger, G Varnaccia, A Borrmann, C Lange, GBM Mensink

Finger JD, Varnaccia G, Borrmann A, Lange C, Mensink GBM (2018)

Journal of Health Monitoring 3(1): 23-30.

DOI 10.17886/RKI-GBE-2018-023

An earlier version of this Fact sheet gave the following incorrect figures on page 23: ‘According to the Global Burden of Disease Study 2016 [2], lack of physical activity in Germany is behind 22.7% of coronary heart disease, 6.3% of stroke, 2.3% of diabetes mellitus, 3.3% of colorectal cancer and 2.0% of breast cancer deaths.’ These figures incorrectly reported the proportion of overall mortality due to the diseases mentioned, irrespective of physical activity. The correct sentence reads: ‘According to the Global Burden of Disease Study 2016 [2], lack of physical activity in Germany is behind 12.3% of coronary heart disease, 7.6% of stroke, 3.1% of diabetes mellitus, 3.4% of colorectal cancer and 1.8% of breast cancer deaths.’

The corrected version of the article is available at DOI 10.17886/RKI-GBE-2018-023.2

